# First report of *Mycobacterium avium* subsp. *hominissuis* in a black howler monkey (*Alouatta caraya*): a study with mycobacterial interspersed repetitive unit–variable number of tandem repeats genetic profiling

**DOI:** 10.2478/jvetres-2025-0027

**Published:** 2025-05-06

**Authors:** Anna Didkowska, Marta Majchrzak, Michał Załuski, Sylwia Brzezińska, Dawid Jańczak, Magdalena Nowak, Wiktoria Tchórz, Ewa Augustynowicz-Kopeć, Katarzyna Matusik, Paweł Parniewski

**Affiliations:** 1 Department of Food Hygiene and Public Health Protection, Institute of Veterinary Medicine, Warsaw University of Life Sciences - SGGW, 02-776 Warsaw, Poland; 2 Institute of Medical Biology, Polish Academy of Sciences, 93-232 Łódź, Poland; 3 The Silesian Zoological Garden in Chorzów, 41-501 Chorzów, Poland; 4 Department of Microbiology, National Tuberculosis Reference Laboratory, National Tuberculosis and Lung Diseases Research Institute, 01-138 Warsaw, Poland; 5 Department of Infectious and Invasive Diseases and Veterinary Administration, Faculty of Biological and Veterinary Sciences, Nicolas Copernicus University in Toruń, 87-100 Toruń, Poland

**Keywords:** atypical mycobacteria, *Giardia intestinalis*, MAH, nonhuman primates, zoonosis

## Abstract

**Introduction:**

Over the past three decades, emerging epidemiological evidence has shown the increasing incidence and prevalence of nontuberculous mycobacteria (NTM). As a result, clinical awareness of the impact these organisms have on both human and animal health has grown.

**Material and Methods:**

Two captive black howler monkeys (*Alouatta caraya*) were experiencing recurrent diarrhoea. Their faecal samples were processed by suspension, decontamination and culture to propagate *Mycobacterium*. Immunochromatography and molecular studies were undertaken to detect parasites. The antimicrobial susceptibility of bacterial isolates was tested using broth microdilution. Additionally, mycobacterial interspersed repetitive unit-variable number of tandem repeats (MIRU-VNTR) typing was performed to assess the *Mycobacterium* pattern.

**Results:**

*Mycobacterium avium* subsp. *hominissuis* was identified and noted to have a novel MIRU-VNTR pattern (22221229). No parasites were detected by immunochromatography, but *Giardia intestinalis* was identified by PCR. This marks the first documented case of co-infection with *Mycobacterium avium* subsp. *hominissuis* and *Giardia intestinalis* in a black howler monkey.

**Conclusion:**

Collecting clinical isolates from infected animals is vital for comparing them with other isolates, including those from the environment, and for re-evaluating their potential as pathogens. Our study is significant within the context of veterinary disease control and the One Health approach.

## Introduction

The *Mycobacterium* genus is a diverse group of both pathogenic and commensal organisms with over 190 distinct species. The species with the most significant impact thus far on human health from clinical, societal and historical perspectives is *Mycobacterium tuberculosis*. However, over the last three decades, emerging epidemiological evidence has highlighted that the incidence and prevalence of other species from the *Mycobacterium* genus referred to as nontuberculous mycobacteria (NTM) are increasing. Given the increased incidence of NTM, clinical awareness of the impact of these organisms on disease states and health has grown. Infection with NTM can interfere with veterinary diagnosis of tuberculosis (TB) and carries some risk of becoming a zoonosis, especially in immunocompromised humans. Bacteria from the *Mycobacteria* genus are also known to be comorbid with other pathogens, especially parasitological ones, including *Giardia intestinalis* (9).

Nontuberculous mycobacteria are increasingly recognised as emerging opportunistic pathogens in both animals and humans (15). In humans, especially in immunocompromised patients, skin contact with NTM bacteria, consumption of contaminated food or even aerosols can result in skin or lung infections and lymphadenitis (15). In the NTM group, the most pathogenic species are bacteria in the *Mycobacterium avium* complex (MAC), and of these, the most clinically important subspecies are *Mycobacterium avium* subsp. *paratuberculosis* (MAP), *Mycobacterium avium* subsp. *hominissuis* (MAH), *Mycobacterium avium* subsp. *avium* (MAA), *Mycobacterium avium* subsp. *intracellulare* and *Mycobacterium avium* subsp. *silvaticum* (15). The *hominissuis* subspecies is also a ubiquitous environmental saprophyte, often isolated from water or soil (25). It is also known to cause mainly lung diseases and lymphadenitis, as well as disseminated MAH infections.

Both MAC and *Mycobacterium tuberculosis* complex bacteria have been confirmed before in Polish zoos (5, 16, 17, 22) and pose a public health risk to visitors. However, there do not appear to be specific studies documenting the isolation of MAH in Poland or in any other country from black howler monkeys (*Alouatta caraya*). Among New World monkeys in zoological gardens, the black howler (*Alouatta caraya*) is the largest. According to the International Union for Conservation of Nature Red List, it is a near-threatened species of which the population reduction is due to anthropomorphic factors.

This study aimed to report the first case of *Mycobacterium avium* subsp. *hominissuis* isolated from two black howler monkeys with recurrent diarrhoea kept in a zoo. The strain was genetically characterised using two methods: the mycobacterial repetitive-unit–variable number of tandem repeats (MIRU-VNTR) method, one of the most reliable methods for genotyping species of the MAC (26); and also insertion sequence (IS) analysis, by which it is possible to determine the types of isolated strains (19). An analysis was conducted to compare the MIRU-VNTR profiles of MAH strains from various databases with this isolate. Minimum spanning tree (MST) analysis was used to illustrate the relatedness among the analysed MAH strains. Additionally, an investigation into drug resistance was carried out. Co-infection with *Giardia intestinalis* and atypical mycobacteria was also observed.

## Material and Methods

### Material

In March 2024, faecal samples were collected in the Silesian Zoological Garden in Chorzów from the enclosure inhabited by two black howler monkeys. One was a 15-year-old female from Apenheul Primate Park (in Apeldoorn in the Netherlands), and the second was a 9-year-old male from Wrocław Zoological Garden (in Wrocław in Poland). The animals arrived at the zoo respectively on 21/09/2023 and 16/11/2023. Until 21/01/2024, a third black howler monkey, a 5-year-old female, was in the enclosure. This animal died of intestinal perforation. Above the monkeys’ cage, there is flying space for a female Green araçari (*Pteroglossus viridis*), which has been kept in the zoo in Chorzów since 27/09/2023 and which came from the Zoological Garden in Łódź in Poland. Some armadillos (*Dasypodidae*) were also housed in the cage for a very short time. The black howler monkeys had suffered from recurrent diarrhoea since arriving at the zoological garden. The only treatment had been the administration of prebiotics.

To reduce stress for the animals and maintain their well-being, the samples were collected with the zookeeper’s help during the routine cleaning of the enclosure. The faeces were collected from a freshly laid plastic tarpaulin shortly after defecation. The material was placed in a sterile container and then transported to the laboratory in refrigerated conditions (8°C). Part of the material was intended for parasitological tests, which were performed within 24 h of collection, and part was frozen at –20°C for further testing.

### Culture of mycobacteria

The faecal samples were processed by suspension, decontamination and culture, according to the World Organisation for Animal Health (WOAH) Terrestrial Manual 2024, Chapter 3.1 (28). Briefly, 1 g of faeces was shaken in distilled water for 30 min at room temperature. Then, the uppermost 5 mL of the faeces suspension was transferred to a tube containing 20 mL of 0.95% 3-hydroxy-2-phenylcinchoninic acid and incubated for 18 h at room temperature. The undistributed sediment was then inoculated into five types of solid media: Stonebrink, Löwenstein–Jensen, Middlebrook and Herrold’s egg yolk (all from Becton Dickinson, Franklin Lakes, NJ, USA), with and without mycobactin. The media were incubated at 37°C for eight months and checked for colonies weekly.

### Antimicrobial susceptibility

Antimicrobial susceptibility was tested using broth microdilution. For this purpose, 96-well SLOMYCO Sensititre titration plates (Thermo Fisher Scientific, Waltham, MA, USA) were used, which allow the simultaneous determination of susceptibility to 14 antibiotics. These plates contain freeze-dried antibiotics in a range of concentrations. A test for *M. avium* was performed as recommended by the Clinical and Laboratory Standards Institute (CLSI) for the bacterium’s susceptibility to clarithromycin, moxifloxacin, amikacin and linezolid (24). At the first stage of the test, an inoculum of a mycobacterial suspension at the optical density of 0.5 McFarland scale was prepared. A 50-μL aliquot of inoculum was transferred to 10 mL of cation-supplemented Mueller–Hinton broth and N-tris(hydroxymethyl)methyl-2-aminoethanesulfonic acid buffer (Thermo Fisher Scientific) with 5% oleic acid–albumin-dextrose-catalase (OADC). One hundred μL of suspension was pipetted into a 96-well titration plate and incubated at 36°C ± 1°C for 7 d (1, 6). Breakpoint to minimum inhibitory concentrations were interpreted and the strain was classified into one of three groups (susceptible (S), intermediate (I) or resistant (R)) following the CLSI criteria (1, 3).

### Isolation of DNA and strain identification

Isolation of DNA from colonies was undertaken using the Genolyse Isolation Kit (Hain Lifescience, Nehren, Germany). Identifying isolated NTM strains was carried out using the GenoType Mycobacterium CM test (Hain Lifescience). Both kits were used according to the manufacturer’s manuals. The GenoType Mycobacterium CM test is qualitative and is based on DNA-strip technology. The procedure consists of the following steps: mycobacterial DNA isolation from the obtained culture, amplification and reverse hybridisation. The presence of bands at control sites (CC, IC and GC) indicates the correct reaction course and the presence of mycobacteria genetic material.

### Insertion sequence 901, IS900 and IS1245 identification

Isolates of DNA were subjected to IS901, IS900 and IS1245 analysis according to the method previously described by Majchrzak *et al*. (19) with modifications. This method is used for the rapid identification of *M. avium* subspecies. A PCR reaction was performed in a total volume of 50 μL containing 20 ng of DNA, 25 μL of Platinum Multiplex PCR Master Mix 2× (Applied Biosystems, Carlsbad, CA, USA) and 50 nM of each primer which was the same as was used by Lalle *at al*. (18). The following conditions were used: an initial denaturation step at 95°C for 15 min; 28 cycles of denaturation at 95°C for 30 s, annealing at 60°C for 90 s and extension at 72C for 1 min; and a final extension step at 72C for 2 min. The reaction was performed using a T3000 thermal cycler (Analytik Jena, Jena, Germany). Electrophoresis was performed at 70 V (2.4 V/cm) until the bromophenol blue dye reached 6 cm from the wells. The gel was then stained in an ethidium bromide solution (0.5 μg/mL) for 10 min and destained in water for another 10 min. It was visualised under UV light using a FluorChem 8800 system with Alpha EaseFC v. 3.1.2 software (AlphaInnotech, San Leandro, CA, USA). A 100 bp Plus DNA Ladder size marker from MBI Fermentas (Vilnius, Lithuania) was used for all analysed IS types. The predicted sizes of the PCR fragments for IS1245, IS900 and IS901 were 427 bp, 389 bp and 262 bp, respectively.

### Identification by MIRU-VNTR typing

The typing method used, MIRU-VNTR, is a powerful technique for analysing mycobacteria genotypes and elucidating possible phylogenetic relationships between strains. The isolate underwent multi-locus variable number of tandem repeats analysis (MLVA) employing the eight variable-number tandem-repeat locus scheme proposed by Thibault *et al*. (26). Primer pairs to amplify the tandem repeat (TR)292, TRX3, TR25, TR47, TR3, TR7, TR10 and TR32 loci were chosen from the work of Cochard *et al*. (7). The PCR procedure was carefully optimised and performed as described by Majchrzak *et al*. (19). The size of each amplicon was determined using BioNumerics software v. 4.6 (Applied Maths, Sint-Martens-Latem, Belgium) to calculate motif repeat number. This number code was then matched against those entered in the MAC-INMV-SSR database (7).

### Clustering analysis by MST

The genotypic data of seven loci (MIRU07, 10, 25, 32, 47, 292 and X3) were analysed. As reported by other investigators, many *M. avium* isolates have not produced PCR products at the TR3 locus. This locus is also known as monomorphic for MAH. Studies involving analysis of seven MIRU-VNTR loci are considered equivalent to the previously reported eight-MIRU-VNTR-locus studies (14). Therefore, we excluded the TR3 locus from further analyses. Clustering analysis of VNTR profiles was performed based on the MST algorithm using the VNTR profiles of the N32 strain and 349 strains isolated from different sources (cattle, pigs, the environment and patients) and different geographical regions (Japan, Finland and France) described in previous reports (14, 31). We used BioNumerics software v. 4.6. A phylogenetic tree was reconstructed by first selecting a categorical coefficient. The priority rule was set so that the type with the highest number of single-locus variants would be linked first, with creation of hypothetical types not permitted (13).

### Parasitological methods

To detect possible concomitant parasitic diseases, a microscopic examination was performed using Faust flotation with zinc sulphate (32). In addition, immunochromatographic tests were carried out to determine the presence of *Giardia intestinalis* and *Cryptosporidium* spp. antigens (Stick Crypto-Giardia; OPERON, Zaragoza, Spain). Afterwards, DNA was isolated using a commercial kit (Genomic AX Stool; A&A Biotechnology, Poland, Gdańsk,), and PCR tests were performed for the presence of DNA of intestinal protozoa: *G. intestinalis* (511 base pair (bp); β-giardin) (18), *Cryptosporidium* spp. (840 bp; 18S small subunit (SSU) ribosomal RNA (rRNA)) (29), *Entamoeba coli* (166 bp; SSU rRNA) (23), *Pentatrichomonas hominis* (339 bp; 18S rRNA) (11). Electrophoresis was performed on a 1.5% agarose gel to reveal the sought genetic material. The positive results were then sent for sequencing to Laboratory of DNA sequencing and synthesis, Institute of Biochemistry and Biophysics, Polish Academy of Sciences in Warsaw.

The material was also tested by using the multiplex PCR method for the presence of *Entamoeba histolytica, Entamoeba dispar* and *Entamoeba moshkovskii* DNA *(ba*, 166 bp; SSU rRNA; 752 bp; SSU rRNA; 580 bp; SSU rRNA), (32). Both, PCR and multiplex PCR tests were performed with commercial mix (StartWarm HS-PCR Mix; A&A Biotechnology, Poland, Gdańsk).

## Results

### Mycobacteria

Numerous nonchromogenic, small, round, cream-coloured colonies of fastidious cells developed on all media types in four to six weeks. The isolated strain was classified as a *Mycobacterium avium* complex subspecies based on the appearance of single colonies on differential media and the GenoType Mycobacterium CM test, and this was confirmed by the IS analysis. The strain showed the presence of IS1245 but not of IS900 or IS901, which allowed its classification as *M. avium* subsp. *hominissuis* (Table 1). A new MIRU-VNTR pattern was identified (22221229) and added to the MAC-INMV-SSR database. The tested strain was found to be susceptible to amikacin and clarithromycin, intermediately susceptible to moxifloxacin, and resistant to linezolid (Table 2).

**Table 1.** Results of mycobacterial interspersed repetitive unit–variable number of tandem repeats (MIRU-VNTR) typing and insertion sequence (IS)901, IS900 and IS1245 identification of a *Mycobacterium avium* strain isolated from black howler monkey (*Alouatta caraya*) faeces

Isolate No.	Strain origin	Number of copies MIRU-VNTR region								Subspecies assignment	IS901	IS900	IS1245
		TR 292	TR X3	TR 25	TR 47	TR 3	TR 7	TR 10	TR 32				
N32	Black howler monkey	2	2	2	2	1	2	2	9	MAH	–	–	+

1 TR – tandem repeat; MAH – *M. avium* subsp. *hominissuis*; + – presence of the tested fragment; – – lack of the tested fragment

**Table 2.** Antibiogram results for the tested *Mycobacterium avium* strain isolated from black howler monkey (*Alouatta caraya*) faeces

Antibiotic	MIC result	Interpretation
Clarithromycin	1	S
Moxifloxacin	8	S
Amikacin	4	R
Linezolid	16	I

1 MIC – minimum inhibitory concentration; S – susceptible; I – intermediately susceptible; R – resistant

### Phylogenetic analysis based on VNTR pattern comparison

**Fig. 1.**
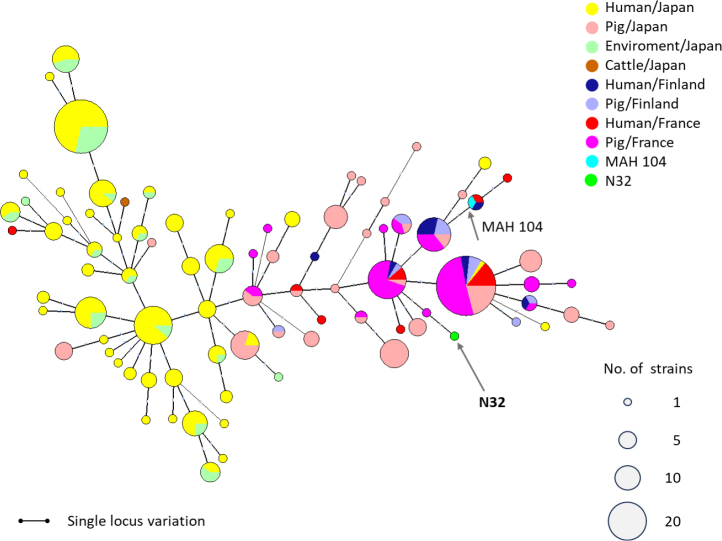
Combined minimum spanning tree analysis based on seven-locus (mycobacterial interspersed repetitive unit-variable number of tandem repeats (MIRU-VNTR) genotyping taking the 07, 10, 25, 32, 47, 292 and X3 loci of *Mycobacterium avium* subsp. *hominissuis* (MAH) strains. The N32 MAH strain isolated from two black howler monkeys with recurrent diarrhoea (N32) and previously published strain data were used for the analysis. Circle sizes are proportional to the number of isolates sharing an identical VNTR pattern. MAH 104 is a reference strain. Lines connecting two types denote single- or double-locus variations with each length

The VNTR eight-locus pattern of strain N32 was determined, and a new pattern was identified. This was the first case of *Mycobacterium avium* subsp. *hominissui*s detected in the black howler monkey; therefore, we decided to compare its VNTR profile with other VNTR patterns available in the databases. We compared our data on the seven MIRU-VNTR loci with data obtained from the seven MIRU-VNTR loci from Japanese (human, porcine, bovine and environmental), French (human and porcine) and Finnish (human and porcine) records and created an MST analysis (Fig. 1). The N32 VNTR pattern was a unique orphan type and was located near French and Finnish isolates (human and porcine) and isolates from Japanese pigs.

### Parasitology

In microscopic examination, the only parasites observed were cysts resembling *Giardia intestinalis* or trichomonas pseudocysts. Because their morphological forms were similar and it was possible for both protozoa to occur in black howler monkeys, it was decided to perform more advanced tests. The immunochromatographic stick test was negative for *G. intestinalis* and *Cryptosporidium* spp. antigens. After performing PCRs and electrophoresis, a positive result was obtained only for the presence of the DNA of *G. intestinalis*. Sequencing resulted in assemblage B (data not shown).

## Discussion

Even though recurrent diarrhoea symptoms and MAC isolation are more typical for MAP (8), our study has shown the isolation of MAH. Considering the clinical symptoms, the considerable growth on all media types and the very careful collection of material (to avoid sampling environmental material), we assume the animals were MAH infected. We have also isolated *G. intestinalis*, which may indicate recurrent coinfection of those two pathogens.

Knowledge of MAH and how it manifests effects in animals remains unclear because there have only been limited studies. Various animal species should be considered potential MAH infection sources (in contrast to the situation with the host-adapted MAP) in order to make MAH surveillance more effective. Previous studies have shown that MAH infection may have tropism for atypical organs and produce atypical symptoms (31). To our knowledge, this is the first case of MAH isolation from the faeces of a symptomatic animal. However, in four free-ranging red deer (*Cervus elaphus hippelaphus*), MAH was isolated from the intestinal tract. The affected animals were suffering from diarrhoea and were emaciated. Post-mortem lesions included enlarged mesenteric lymph nodes and a thickened intestinal wall (10).

Linezolid and moxifloxacin are among the recommended antibiotics for treating MAC infections in humans (4) and are used as second-line drugs. They are used when first-line drugs (clarithromycin and amikacin) have not worked (2). Similar antibiotic resistance to that observed in the MAH from the black howler monkeys has been previously described in *M. avium* strains from human patients (30). Also, MAH biofilm was resistant to exposure to ampicillin, moxifloxacin, rifampin and trimethoprim-sulphamethoxazole (21). In non-human mammals, *M. avium* subsp. *avium* isolated from wild Eurasian otters (*Lutra lutra*) showed similar susceptibility to antibiotics (24) as in our case. Interestingly, the MAH strain isolated from the black howler faeces was susceptible to first-line drugs but intermediately susceptible and resistant to linezolid and moxifloxacin. This underlines the bacterium’s zoonotic potential and leads to challenges in the treatment of infections with it.

Many previous studies have focused on analysing the genetic diversity and population structure of MAH, and the relationship between genotypes and infection prognosis. Notably, a high similarity has been reported between MAH isolates from pigs with disseminated disease and clinical human sputum samples in European countries, which contrasts with findings from Japan. In this study, we aimed to assess the genetic relatedness of the N32 strain isolated from the two black howler monkeys to other MAH strains by comparing it with registered strain data using VNTR typing. The novel VNTR genotype N32 was classified as a unique orphan type, positioned close to a cluster of strains from European patients and pigs. The true genetic population structure of MAH may be more extensive than currently known because the data available from various sources are limited. In addition, our case is a special case because the animal had been kept in a zoo for many years. Therefore, further collection of genotypic data on MAH strains from diverse origins will be essential for a better understanding of their transmission.

*Giardia intestinalis* is a common gastrointestinal parasite. Most often, invasion in animals, including nonhuman primates (NHP), is asymptomatic (20). However, it can also cause diarrhoea, vomiting, abdominal cramps and malabsorption, which is especially dangerous for young and weakened individuals (12). So far, co-infections with *G. intestinalis* and MAH have not been documented in NHPs. However, they have been reported in chinchillas (*Chinchilla lanigera*) with multisystem, multifocal granulomatous inflammation, which also affected the gastrointestinal tract (1). No more information is available regarding atypical mycobacteria in co-infections, but an analysis of the co-pathogenesis between *M. tuberculosis* and *G. intestinalis* in humans has been performed. It was found that the risk of tuberculosis was higher in patients with *G. intestinalis*, and patients with tuberculosis were more easily infected with *G. intestinalis* than those without pulmonary tuberculosis (27). Therefore, it is possible that in the case of the black howler monkeys we studied, there was disease enhancement between these pathogens, generally worsening the animal’s condition.

Our study is important in the contexts of both veterinary disease control and the One Health approach. Collecting clinical isolates from infected animals is vital for comparing them with other isolates, including those from the human environment, and for re-evaluating their potential as pathogens.

## Conclusion

This study documents the first known case of *Mycobacterium avium* subsp. *hominissuis* in black howler monkeys (*Alouatta caraya*), which presented with recurrent diarrhoea. A unique MIRU-VNTR pattern was identified for the isolated strain, contributing to the genetic characterisation of *Mycobacterium avium* subsp. *hominissuis*. The monkeys were also found to be co-infected with *Giardia intestinalis*, highlighting the potential for opportunistic infections in nonhuman primates. The increasing incidence of nontuberculous mycobacteria in both humans and animals emphasises the need for awareness and monitoring, particularly in zoological settings where human–animal interactions occur. This study underscores the importance of collecting clinical isolates from animals in veterinary disease control and reinforces the One Health approach that recognises the interconnectedness of human, animal and environmental health.
